# Screening and diagnosis of gestational diabetes mellitus – relevance to low and middle income countries

**DOI:** 10.1186/s40842-016-0031-y

**Published:** 2016-06-01

**Authors:** B. Bhavadharini, R. Uma, P. Saravanan, V. Mohan

**Affiliations:** 1 Madras Diabetes Research Foundation & Dr. Mohan’s Diabetes Specialities Centre WHO Collaborating Center for Non-Communicable Disease Prevention and Control, 4, Conran Smith Road, Gopalapuram, Chennai, 600 086 India; 2 Seethapathy Clinic and Hospital, Chennai, India; 3 Warwick Medical School, University of Warwick, Coventry, UK

**Keywords:** Screening, IADPSG, Gestational diabetes mellitus, Low middle income countries, WINGS, India, Asian Indians, South Asians

## Abstract

Gestational diabetes mellitus (GDM) is one of the most common metabolic complications of pregnancy. Ever since the first systematic evaluation of the oral glucose tolerance test by O’Sullivan and colleagues was carried out in 1964, there has been controversy with respect to the optimal screening and diagnostic criteria to detect GDM. The recently proposed International Association of Diabetes and Pregnancy Study Groups (IADPSG) criteria for GDM has found fairly widespread acceptance, but it is still debated by several societies. This review intends to provide an overview of the evolution of the screening and diagnostic criteria for GDM. Debatable issues regarding optimal screening strategies, especially in the low resource settings of low and middle income countries are highlighted. The recent Women in India with GDM Strategy (WINGS) project carried out in Chennai, India tried to develop a Model of Care for GDM suitable for resource constrained settings. The findings related to screening and diagnosis of GDM based on WINGS are also highlighted in this review. Based on the WINGS experience we believe that despite the constraints in low and middle income countries at the present time, the IADPSG criteria appears to be the best. This will also help to bring out a uniform criteria for screening and diagnosis of GDM worldwide.

## Background

The criteria for diagnosing diabetes outside of pregnancy, has evolved over time and have been largely accepted by major diabetes organizations worldwide. However, the screening and diagnosis of gestational diabetes mellitus (GDM) continues to be a contentious issue. Notwithstanding decades of research and several international workshops devoted to GDM, there is still no consensus among international bodies on a uniform global approach to screening and diagnosis of GDM [1]. Indeed, guidelines for GDM screening and diagnosis vary among countries and between major societies worldwide. Often, even within a country, there is lack of consensus between the diabetes and obstetric societies, with each of them recommending a different approach [2].

The lack of consensus regarding the screening and diagnostic criteria for GDM means that different sets of women would be identified as having GDM by the different criteria. The guidelines used depend on several factors like the availability of infrastructure, cost considerations and patient convenience. Poor dissemination of information and availability of resources could be some of the reasons for these conflicting guidelines used in different settings.

### Controversies in screening and diagnosis of GDM

The terms “screening” and “diagnosis” are often used interchangeably. A screening test for GDM identifies women at greater or lower risk of GDM depending on a particular glucose threshold. Those exceeding the threshold in the screening test should then proceed to a definitive diagnostic test, which provides a definitive answer to the presence or absence of GDM.

There has been a lot of controversy in literature with respect to almost all aspects of screening for GDM. These include many fundamental questions such as why and how to screen for GDM, universal screening or selective screening, one step procedure or two step procedure, early (first trimester) screening or second trimester screening etc. A detailed discussion on each of these points would be beyond the scope of this article. Hence we will restrict ourselves, in the first part of this review to the historic evolution of the various diagnostic criteria over time. The second part of the article will focus on the challenges in screening and diagnosis of GDM in low resource settings, specifically drawing experience from the Women in India with GDM Strategy (WINGS) [3, 4] project which was recently completed in southern India.

### Historical evolution of diagnostic criteria for GDM

#### The O’Sullivan and Mahan criteria

First proposed in 1964, the O’Sullivan and Mahan [5] criteria formed the basis for the majority of criteria that subsequently evolved. O’Sullivan and Mahan suggested the use of a 50 g 1 h glucose challenge test (GCT) to screen for GDM followed by a diagnostic (confirmatory) test in those who were GCT positive (i.e., 1 h post glucose load exceeds 140 mg/dl) using 100 g 3-h oral glucose tolerance test (OGTT). These criteria were based on a series of 752 women who underwent OGTT during pregnancy. These thresholds (Table 1) were based on venous whole blood samples analysed by the Somogyi-Nelson method and were based on their ability to predict maternal diabetes subsequent to pregnancy. They came into widespread use in the late 1970s.

**Table 1:** Various criteria proposed for diagnosing GDM based on fasting OGTT

Criteria	Year proposed	Approach	Glucose load (g)	Glucose threshold mg/dl (mmol/l)			
				Fasting	1 h	2 h	3 h
O’Sullivan & Mahan	1964	2 step	100	90 (5.0)	165 (9.2)	145 (8.1)	125 (6.9)
National Diabetes Data Group (NDDG)	1979	2 step	100	105 (5.8)	190 (10.6)	165 (9.2)	145 (8.1)
Carpenter & Coustan	1982	2 step	100	95 (5.3)	180 (10.0)	155 (8.6)	140 (7.8)
World Health Organization (WHO)	1999	1 step	75	126^a^ (7.0)	–	140 (7.8)	–
American Diabetes Association (ADA)	2004	2 step	100	95 (5.3)	180 (10.0)	155 (8.6)	140 (7.8)
Latin American Diabetes Association (ALAD)^b^	2008	2 step	75	100 (5.5)	–	140 (7.8)	–
International Association of Diabetes and Pregnancy Study Groups (IADPSG)	2010	1 step	75	92 (5.1)	180 (10.0)	153 (8.5)	–
World Health Organization 2013 criteria (revised, same as IADPSG)	2013	1 step	75	92 (5.1)	180 (10.0)	153 (8.5)	–
National Institute for Health and Care Excellence (NICE)	2015	1 step	75	101 (5.6)	–	140 (7.8)	–

Adopted from (Vandorsten et al., 2011) [15]

Values in parenthesis are in mmol/l

^a^Later this fasting value was dropped

^b^Criteria for the diagnosis of gestational diabetes in selected countries of the Americas. Final report of the Pan American Conference on Diabetes and Pregnancy [32]

### The National Diabetes Data Group and the Carpenter & Coustan criteria

Over the years, there was a universal change in laboratory techniques in glucose assay methods. The less specific Somogyi-Nelson method was replaced by enzyme based assays which were now performed on plasma, rather than whole blood. Thus the National Diabetes Data Group (NDDG) [6] in 1979 proposed thresholds that were approximately 15 % higher than the original O’Sullivan and Mahan cut points.

Subsequently in 1982, Carpenter and Coustan introduced a correction factor and modified the O’Sullivan and Mahan criteria empirically by adjusting for the differences in assay methods. This was later validated by Sacks et al. [7] and thus the famous Carpenter and Coustan criteria for GDM came into existence and soon became widely accepted [8].

### American Diabetes Association (ADA) criteria for GDM

The ADA endorses the Carpenter and Coustan criteria and recommends that women with high risk of GDM (Table 2) undergo glucose testing as early as possible during pregnancy [9]. A fasting plasma glucose (FPG) ≥126 mg/dl (7.0 mmol/l) or a random blood glucose ≥200 mg/dl (11.1 mmol/l) is diagnostic of pregestational diabetes and has to be confirmed early in pregnancy. The ADA recommends that the testing for GDM at 24–28 weeks be done either by one step approach using a 100 g OGTT or by two step process, with an initial test using 50 g GCT followed by the diagnostic OGTT using 100 g glucose load [9].

**Table 2:** Risk factors to screen for GDM [Recommended by the American Diabetes Association (ADA) [9]]

• Age ≥25 years
• BMI ≥25 kg/m ^2^ in Americans and BMI ≥23 kg/m ^2^ in Asian Americans
• High risk ethnic groups – South Asian, Aboriginal, Hispanic
• Previous history of GDM
• Family history of type 2 diabetes
• History of poor obstetric outcomes – congenital malformations, still birth etc.

### The World Health Organization (WHO) 1999 criteria for GDM

In 1999, a WHO Expert Group recommended that pregnant women who met the WHO criteria for impaired glucose tolerance (IGT) in the non-pregnant state, i.e., 140 mg/dl (7.8 mmol/l) be classified as GDM. However, they had also included a FPG ≥126 mg/dl (7.0 mmol/l) [10]. Soon it was realized that the fasting ≥126 mg/dl (7.0 mmol/l) level was inappropriate as it is diagnostic of diabetes in the non-pregnant state. Hence it was later dropped and subsequent studies used only the 2 h post glucose cut point of 140 mg/dl (7.8 mmol/l) for diagnosis [11, 12]. In 2013, the WHO essentially dropped its 1999 criteria and endorsed the International Association of Diabetes and Pregnancy Study Groups (IADPSG) criteria that are discussed below.

### Drawbacks of the earlier criteria

Each of the above criteria had some drawback or the other. The O’Sullivan and Mahan criteria were derived mathematically and were validated for future diabetes in the mother rather than the outcomes of pregnancy. The WHO 1999 criteria, also predicted future development of type 2 diabetes in mothers and were simply based on criteria for IGT used in non-pregnant adults without taking into account the changes in carbohydrate metabolism brought about by pregnancy. The ADA criteria was based on older data. Thus it was clear that newer, evidence based, criteria validated by their ability to predict adverse pregnancy outcomes in both mother and the baby, were urgently needed.

### The Hyperglycemia and Adverse Pregnancy Outcomes (HAPO) study

To deal with the wide disparity in diagnostic testing and to clarify the unanswered questions regarding the association of glucose with risks of adverse pregnancy outcomes, the Hyperglycemia and Adverse Pregnancy Outcomes (HAPO) study was planned. HAPO was a large multi national and multi center study, which included over 23,000 pregnant women [13]. OGTTs were administered between 24–32 weeks gestation in a heterogenous and an ethnically diverse group of pregnant women. Results of the HAPO study showed a linear relationship between glucose values and several primary (umbilical cord-blood C-peptide level, birth weight, neonatal hypoglycemia, and rate of cesarean delivery) and secondary outcomes (fetal adiposity, preeclampsia, birth trauma, admission to NICU, shoulder dystocia). These were directly related to the levels of fasting as well as independently, to the 1 h, and 2 h post-challenge glucose values. The major conclusion of the study was that the risk of adverse pregnancy outcomes continuously increased as a result of elevated maternal fasting or post load glycemia measured at 24–28 weeks gestation.

### International Association of Diabetes and Pregnancy Study Groups (IADPSG) criteria

On the basis of the HAPO data, the International Association of Diabetes and Pregnancy Study Groups (IADPSG) panel suggested a single step 75 g OGTT to be done in all pregnant women at 24–28 weeks of gestation [14]. Glucose values which yielded an odds ratio (OR) of 1.75, i.e., which had a 75 % increase in risk of developing 3 combined primary outcomes (more than 90^th^ centile of birth weight, total body adiposity based on skin folds and cord blood C-peptide levels). The diagnosis was to be made if any one of the values for fasting plasma glucose, 1-h glucose, or 2-h glucose equaled or exceeded the diagnostic threshold as shown in Table 3.

**Table 3:** IADPSG criteria for diagnosis of GDM and overt diabetes in pregnancy [14]

First prenatal visit	Measure FPG, HbA1c or RPG	Overt diabetes if, FPG ≥ 126 mg/dl (7.0 mmol/l) Or random plasma glucose ≥ 200 mg/dl (11.1 mmol/l) Or HbA1c ≥ 6.5 % GDM if, FPG ≥ 92 mg/dl (5.1 mmol/l) but <126 mg/dl (7.0 mmol/l)
If the test is normal in the first prenatal visit, test for GDM during 24–28 weeks		
24–28 weeks of gestation	75 g OGTT	Pre existing diabetes if FPG ≥ 126 mg/dl (7.0 mmol/l) GDM if, FPG ≥ 92 mg/dl (5.1 mmol/l) 1 h ≥ 180 mg/dl (10.0 mmol/l) 2 h ≥ 153 mg/dl (8.5 mmol/l)

IADPSG criteria also recommended the threshold for the diagnosis of GDM as well as overt diabetes at first trimester. Following the IADPSG recommendations, several other guidelines now recommend screening in early trimester to detect overt diabetes especially in high-risk groups [9, 15–17].

### Controversy surrounding GDM diagnosis in early pregnancy

The IADPSG recommends a threshold of ≥ 92 mg/dl (5.1 mmol/l) [14] to diagnose GDM in early pregnancy. This was not based on any data, but on a consensus to use the same FPG cut off of 92 mg/dl which is used at 24–28 weeks to be used at any point of time during pregnancy to diagnose GDM. It has therefore been a subject of intense debate. However in support of this, a recent study from Australia has shown that high risk women, diagnosed with GDM at less than 12 weeks of gestation had adverse pregnancy outcomes similar to that seen in women with pre existing diabetes [18]. This justifies the diagnosis of GDM early in pregnancy.

In contrast, studies from Italy and China have shown that using such low FPG cut offs is inappropriate to diagnose GDM in early pregnancy [19, 20]. Corrado et al. [19] showed no correlation between OGTT at 24–28 weeks and the FPG in early pregnancy. Zhu et al. [20] showed that not all women with FPG ≥92 mg/dl (5.1 mmol/l) in the first trimester developed GDM during 24–28 weeks. These authors have shown that FPG at first trimester was not consistent with FPG at 24–28 weeks since less than one third of women maintained ≥92 mg/dl (5.1 mmol/l) between first trimester and 24–28 weeks. However, they do accept that doing an FPG at first visit could be useful in diagnosing undiagnosed overt diabetes.

Because of the low fasting cut point, the IADPSG criteria has been reported to result in increased prevalence rates of GDM and this increases the burden on the health care system in many countries [21–23]. Very few studies have addressed the cost effectiveness of this criteria. Uncertainties about relevance of treating milder GDM cases and lack of clinical trials addressing these issues are other criticisms of the IADPSG criteria [24].

In response to these objections, the members of the IADPSG council in a recent publication, suggested that the identification of GDM in early pregnancy using FPG ≥92 mg/dl (5.1 mmol/l) was not justified due to insufficient evidence. They recommend identifying only women with HbA1c of ≥5.9 % in early pregnancy, who were at risk of developing adverse pregnancy outcomes [25].

### Adoption of IADPSG criteria by various scientific bodies

Though the IADPSG recommendations for first trimester diagnosis of GDM have been questioned, their recommendations for diagnosing GDM at 24–28 weeks are based on evidence that correlate maternal glucose concentrations to fetal outcomes. These recommendations also reflect a consensus view and have been accepted by a large number of scientific bodies. In 2011, the ADA endorsed the IADPSG criteria [26]. ADA recommended that all pregnant women not known to have prior diabetes undergo a 75 g OGTT at 24–28 weeks. Although the thresholds recommended by IADPSG are only minimally different from the ADA criteria, the prevalence of GDM would still be higher, if IADPSG criteria were used. This is because while the ADA uses 100 g OGTT, requires 2 elevated readings, the IADPSG uses the 75 g load and requires only one abnormal value.

Duran et al. [27], prospectively studied two groups of women: – one group identified as GDM by the Carpenter and Coustan criteria and the other by the IADPSG criteria. Both groups after receiving the same treatment and follow up, were evaluated for health outcomes. This study, one of the first to address the economic benefits of the one step IADSPG criteria, had 4 major conclusions: One, applying IADPSG criteria was associated with decrease in adverse pregnancy outcomes like preeclampsia, cesarean deliveries, macrosomia and LBW, neonatal intensive care unit admission, compared to the Carpenter and Coustan criteria. Second, IADPSG criteria identifies women who had milder GDM, who were considered to be normal by Carpenter and Coustan criteria, thereby increasing the prevalence rates. Third, IADPSG criteria did not lead to overtreatment of GDM as patients needing insulin therapy were not different from the other group of GDM identified by Carpenter and Coustan criteria. Lastly, IADPSG criteria did not increase the health care costs since with the improvement in pregnancy outcomes, rate of cesarean deliveries and number of NICU admissions were reduced. Results from a study in China [28], comparing the IADPSG and the Carpenter and Coustan criteria reported that IADPSG criteria showed better pregnancy outcomes (lower primary cesarean section and lesser neonatal morbidity) than the Carpenter & Coustan criteria, but at the expense of increased prevalence of GDM.

As already mentioned, in 2013, the WHO also accepted the IADPSG criteria [29]. Other organizations like the Endocrine Society [17] and the Australian Diabetes in Pregnancy Society (ADIPS) [30] and recently the International Federation of Gynecology and Obstetrics (FIGO) [31] have also accepted the IADPSG criteria.

In 2013, the National Institute of Health (NIH) convened a consensus meeting on GDM to review the recommendations by IADPSG criteria. The panel report stated that there was insufficient evidence to support the single step approach proposed by IADPSG and therefore recommended that the 2 step approach of screening with 1 h 50 g GCT followed by 100 g GTT as a diagnostic test for GDM, be continued [15]. The American College of Obstetricians and Gynecologists also support the 2 step approach in their guidelines released in 2013 [16].

### Criteria for GDM diagnosis in South and Central America and Europe – trending towards IADPSG criteria

While some Latin American countries have adopted the IADSPG criteria, many have incorporated local modifications to the criteria due to practical difficulties in implementing in low resource settings. A recent compilation of information from the Pan American Conference on Diabetes and Pregnancy Report 2015 [32] elucidated much needed data on the different criteria followed in different South and Central American countries. The Argentine Diabetes Society (SAD) based on a recent multicentric study which compared the IADPSG criteria and the Latin American Diabetes Association (ALAD) criteria (Table 1) showed prevalence of GDM to be 10.4 % by ALAD as against 26.7 % by IADPSG criteria, and hence recommends following the ALAD criteria for GDM diagnosis. Other countries like Guyana, Guatemala, Nicaragua, Colombia and Peru have adopted the IADPSG criteria. However, not all of them follow the IADPSG criteria as a one step process as recommended, but as two step process following an abnormal GCT.

Similar methods are followed in some parts of Europe, where Germany for example follows a 75 g OGTT of IADPSG criteria following a 50 g GCT [33]. It is to be noted however that this method of GCT followed by IADPSG criteria is currently not validated. Ireland, France and some parts of Belgium have adopted the IADPSG criteria [33], but only in high risk individuals.

With the aim of developing a consensus for GDM screening and diagnosis across Europe, the European Board and College of Obstetrics & Gynecology (EBCOG) in association with the European Association for the Study of Diabetes (EASD) have proposed screening for overt diabetes at first antenatal visit using cut off for diabetes outside of pregnancy and to perform GDM screening at 24–28 weeks with a 2 h 75 g OGTT as recommended by the IADPSG. Due to lack of evidence on classifying GDM using FPG ≥92 mg/dl (5.1 mmol/l) in first trimester or first antenatal visit, the EBCOG states that no clear recommendations can be made on diagnostic criteria for GDM in early pregnancy [33].

### National Institute for Health and Care Excellence (NICE) 2015 criteria

Initially the NICE guidelines had recommended the same criteria for GDM diagnosis as the WHO 1999 criteria. In 2015, NICE [34] modified the WHO 1999 criteria and on the basis of health economic analysis, revised their guidelines recommending a fasting cut points of 101 mg/dl (5.6 mmol/l) in addition to the 2 h post glucose value of 140 mg/dl (7.8 mmol/l). At the first antenatal booking appointment, screening for GDM is done through risk factor based assessment. Women with any one of the risk factors are then advised to undergo testing for GDM using the 2 h 75 g OGTT and diagnosis is made as per the thresholds mentioned in Table 2. In women with a previous history of GDM, NICE 2015 recommends a 75 g 2 h OGTT immediately after booking, whether in the first or second trimester and a further 75 g 2 h OGTT at 24–28 weeks, if the test was normal in the earlier visit.

The main difference between IADPSG criteria and NICE 2015 guidelines is that while IADPSG criteria is based on reducing risk of harm to both mother and baby, the NICE 2015 is based on reducing the average unit costs for selected adverse outcomes using health economic modelling which compared the cost effectiveness of NICE risk factor based screening with universal screening. The analysis showed that the incremental cost effectiveness ratios (ICER) in risk factor selected population using NICE 2015 criteria was in the range of £20,000 to £30,000 per quality adjusted life year (QALY), as compared to an ICER of £47,000 in the subset without risk factors. The model therefore did not support change to universal testing as recommended by IADPSG [35]. However, while cost effectiveness is important, it is also important to consider the burden of complications and its effect on emotional and psychological wellbeing, which cannot be measured in economic terms alone [36].

### Challenges in screening and diagnosis of GDM in low resource settings

Notwithstanding the scientific validity of any guideline, there are constraints of applying these criteria in low and middle-income countries (LMIC) where resources are poor. In some remote rural areas, lack of access to a standardized laboratory and resources for performing the test are huge challenges that needs to be addressed. Often, lack of trained phlebotomists to collect venous blood samples, as required by most guidelines, pose a serious challenge in ensuring universal screening. In many rural locations, pregnant women have to travel long distances to meet the doctor and women do not routinely attend antenatal check up in fasting state unless and otherwise informed earlier. Hence bringing back women to undergo the test in the fasting state could be a challenge. Table 4 and 5 summarizes the health care system barriers and the patient related barriers to screening for GDM in low resource settings.

**Table 4:** Challenges in screening for GDM in low resource settings – Health care system barriers

Health care system barriers	
Lack of trained health care professionals	
Lack of trained phlebotomists	
Lack of diagnostic facilities and standardized laboratories	
Storage and transport of blood samples

**Table 5:** Challenges in screening for GDM in low resource settings – Patient related barriers

Patient barriers	
Coming for check up in the fasting state	
Late contact with health care system	
Lack of awareness about GDM and its complications	
Distance to the primary health center/ higher centers	
Undergoing the OGTT in the fasting state	

### Diabetes in Pregnancy Study Group India (DIPSI) criteria

To address some of these barriers, the Diabetes in Pregnancy Study Group of India (DIPSI) introduced simplified guidelines for screening and management of GDM in India. DIPSI recommends that an OGTT can be performed using a 75 g glucose load, irrespective of whether the woman is fasting or not, and a 2-h venous plasma glucose (VPG) value of 140 mg/dl (7.8 mmol/l) be used as the single step definitive, screening and diagnostic test for GDM. These guidelines were based on a single-centre study [37] from southern India which reported a 100 % sensitivity and 100 % specificity for this cut point compared to the WHO 1999 criteria which also uses the same cut point of 140 mg/dl [7.8 mmol/l] but with the OGTT done in the fasting state.

In a resource limited setting like India, where nearly 70 % of the population lives in rural settings [38], DIPSI felt that there was a need to develop a simple and economical method of diagnosing GDM. The DIPSI criteria, because of its sheer simplicity, has been widely accepted and used in many parts of India and Sri Lanka [39] and other South Asian countries. However, as shown below, recent studies have not been able to reproduce the near perfect sensitivity and specificity of the non fasting DIPSI test compared to the fasting OGTT.

### The WINGS project

Acknowledging the challenges for screening and management of GDM in low resource settings, the International Diabetes Federation (IDF) with an unrestricted support from Abbott Fund, launched the Women in India with GDM Strategy (WINGS) [3] project in collaboration with the Madras Diabetes Research Foundation and the project was carried out in Chennai in southern India. The aim of the project was to improve the health outcomes of women with GDM and their babies and to strengthen the capacity of health facilities in low resource settings. The project helped the development of a Model of Care for GDM, which was then tested whether it was effective and feasible for implementation in resource-constrained settings [4].

One of the aims of the WINGS project was to find a cost effective screening strategy and the logical step was to try to replicate the DIPSI results because if a non fasting OGTT can be used it addresses several of the barriers mentioned in Table 4 and 5.

Hence as one of the first objectives, the WINGS project compared the non fasting OGTT with a standard fasting OGTT for screening for GDM using both IADPSG and the WHO 1999 criteria. The WINGS project showed that the non-fasting OGTT had very low sensitivity (22.6 %) compared to both the IADPSG criteria as well as the WHO 1999 criteria (27.7 %) [40]. The specificity of the DIPSI criteria was however found to be high - 97.8 and 97.7 % compared to the IADPSG and WHO 1999 criteria respectively. Another study from Delhi, in north India also confirmed the low sensitivity of the DIPSI criteria and concluded that it would miss a substantial number of GDM patients and these authors suggested that the IADPSG criteria is most suitable for screening for GDM in India [41]. A study from Sri Lanka, also confirmed that the sensitivity of the DIPSI criteria was low (40.6 %) [42]. Indeed, the low sensitivity of the non-fasting OGTT was shown several decades ago by Coustan et al. [43]. The physiology of the non-fasting state, can explain the low sensitivity of the non-fasting OGTT. After a woman consumes a carbohydrate meal, blood glucose levels increase, thereby stimulating insulin release. When a glucose load is given at this point, blood glucose levels are blunted since insulin levels are already elevated. The sensitivity of the test therefore drops dramatically as shown in above mentioned studies. Hence the WINGS project supports all international guidelines, which recommend that the diagnostic OGTT should be done after an overnight fast.

As pointed out earlier, in low resource settings, obtaining venous plasma glucose samples is sometimes next to impossible due to shortage of trained phlebotomists in addition to limited access to standardized laboratories to carry out venous glucose estimations. However, currently almost all criteria for GDM require venous plasma samples for diagnosis of GDM. The question then arises, what if it is impossible to obtain a venous blood sample? In such situations, if screening for GDM is to be done at all, the only alternative would be to use a hand held blood glucose meter to perform capillary blood glucose (CBG) testing. The advantage of using the CBG is that even lay people can be trained to do the screening as it serves as a portable, point of care device for screening. Moreover, obtaining a finger prick sample is minimally invasive when compared with venous blood draw and hence more acceptable to the subjects [44]. Currently however, the use of CBG for diagnosis of GDM is not recommended by any association or guideline. The second objective of the WINGS project was to compare CBG estimations using hand held glucose meter and venous plasma glucose (VPG) estimations. The results showed that the CBG had a low sensitivity when compared with VPG. Moreover, additional women, not identified by the IADPSG criteria to have GDM were picked up by the CBG. In addition, the receiver operating characteristic curve with different 2 h CBG cut points showed that a 2 h CBG cut point of 126 mg/dl (7.0 mmol/l) had the optimum sensitivity and specificity of 70.8 and 63 % compared to the IADPSG criteria. If this cut point is lowered to 120 mg/dl (6.6 mmol/l), the sensitivity improved to 78.3 %, and if lowered to 110 mg/dl (6.1 mmol/l), it improved to 92.5 %. However, 50.1 and 68.9 % of women respectively, have to be referred for the definitive diagnostic OGTT using VPG [45]. Hence the WINGS project concluded that while the CBG cannot effectively replace VPG for diagnosis of GDM it can perhaps be used as an initial screening test (similar to the older GCT test) in resource-constrained settings.

In summary, the WINGS project suggests that ideally, and whenever feasible, a single step 75 g OGTT using the IADPSG criteria should be done in the fasting state and that VPG still remains the gold standard. However, in resource limited settings, especially in the rural areas of developing countries where getting all pregnant women to come in a fasting state may be difficult, the well validated two step procedure using the 50 g OGCT in the non-fasting state as the screening test followed by fasting OGTT for a definitive diagnosis can be continued. In situations where obtaining a venous sample is impossible, the CBG could be used as an initial screening test by maximizing sensitivity by lowering the 2 h cut points. Those who screen positive, could then be referred to higher centres for the definitive diagnostic OGTT in the fasting state, using VPG.

Where does this leave the DIPSI criteria which is quite widely used in India? As the specificity of DIPSI test is good, women diagnosed by the DIPSI criteria most likely, do have GDM. However, a substantial number of women who have GDM are likely to be missed. Our suggestion would be that in situations where all pregnant women cannot be referred in the fasting state for a diagnostic OGTT, the DIPSI non fasting test can be done as an initial screening test but with a lower cut point to maximize the sensitivity. Those who screen positive in this screening test, can then be referred for a definitive OGTT done in the fasting state. However, this needs to be tested for validation as a two step technique by future studies.

## Conclusions

The decision on the best strategy for screening and diagnosis should be made based on cost and availability of the locally existing health facilities. Ultimately it is ideal that all countries should use criteria that are internationally accepted. All things considered, the IADPSG criteria seems to be the most suitable at present for screening and diagnosis of GDM. However there is a need for studies on cost effectiveness of IADPSG criteria and its applicability especially in resource constrained regions of the world. Future research on GDM should be undertaken with greater international collaboration, to address the existing gaps in knowledge on this very important public health problem.

## Abbreviations

CBG. capillary blood glucose; DIPSI. Diabetes in Pregnancy Study Group India; GDM, Gestational diabetes mellitus; HAPO. Hyperglycemia and Adverse Pregnancy Outcomes; IADPSG. International Association of Diabetes and Pregnancy Study Groups ; VPG. venous plasma glucose; WINGS. Women in India with GDM Strategy

## References

[1] Holt RI, Coleman MA, McCance DR (2011). The implications of the new International Association of Diabetes and Pregnancy Study Groups (IADPSG) diagnostic criteria for gestational diabetes. Diabet Med.

[2] Leary J, Pettitt DJ, Jovanovic L (2010). Gestational diabetes guidelines in a HAPO world. Best Pract Res Clin Endocrinol Metab.

[3] http://www.idf.org/women-india-gdm-strategy-wings. Accessed 25 Dec 2015.

[4] Kayal A, Mohan V, Malanda B, Anjana RM, Bhavadharini B et al. Women in India with GDM Strategy (WINGS) – Methodology and development of Model of Care for GDM (WINGS 4). IJEM. 2016. In press. 10.4103/2230-8210.189230 PMC5040055 27730085

[5] O’Sullivan J, Mahan C (1964). Criteria for the oral glucose tolerance test in pregnancy. Diabetes.

[6] National Diabetes Data Group (1979). Classification and diagnosis of diabetes mellitus and other categories of glucose intolerance. Diabetes.

[7] Sacks DA, Abu-Fadil S, Greenspoon JF, Fotheringham N (1989). How reliable is the fifty-gram, one-hour glucose screening test. Am J Obstet Gynecol.

[8] Carpenter MW, Coustan DR (1982). Criteria for screening tests for gestational diabetes. Am J Obstet Gynecol.

[9] American Diabetes Association (2016). 2. Classification and diagnosis of diabetes. Diabetes Care.

[10] World Health Organization (1999). Definition, diagnosis, and classification of diabetes mellitus and its complications: report of a WHO consultation.

[11] Seshiah V, Balaji V, Balaji MS, Paneerselvam A, Arthi T, Thamizharasi M (2007). Gestational diabetes mellitus manifests in all trimesters of pregnancy. Diabetes Res Clin Pract.

[12] Seshiah V, Balaji V, Balaji MS, Sanjeevi CB, Green A (2004). Gestational diabetes mellitus in India. J Assoc Physicians India.

[13] Metzger BE, Lowe LP, Dyer AR (2008). HAPO study cooperative research group. Hyperglycemia and adverse pregnancy outcomes. N Engl J Med.

[14] Metzger BE, Gabbe SG, Persson B (2010). International association of diabetes and pregnancy study groups consensus panel. International association of diabetes and pregnancy study groups recommendations on the diagnosis and classification of hyperglycemia in pregnancy. Diabetes Care.

[15] Vandorsten JP, Dodson WC, Espeland MA, Grobman WA, Guise JM, Mercer BM (2013). NIH consensus development conference: diagnosing gestational diabetes mellitus. NIH Consens State Sci Statements.

[16] Committee on Practice Bulletins – Obstetrics (2013). Practice bulletin No 137: gestational diabetes mellitus. Obstet Gynecol.

[17] Blumer I, Hadar E, Hadden DR (2013). Diabetes and pregnancy: an endocrine society clinical practice guideline. J Clin Endocrinol Metab.

[18] Sweeting AN, Ross GP, Hyett J, Molyneaux L, Constantino M, Harding AJ, Wong J (2016). Gestational diabetes mellitus in early pregnancy: evidence for poor pregnancy outcomes despite treatment. Diabetes Care.

[19] Corrado F, D’Anna R, Cannata ML, Interdonato ML, Pintaudi B, Di Benedetto A (2012). Correspondence between first-trimester fasting glycaemia, and oral glucose tolerance test in gestational diabetes diagnosis. Diabetes Metab.

[20] Zhu WW, Yang HX, Wei YM, Yan J, Wang ZL, Li XL, Wu HR, Li N, Zhang MH, Liu XH, Zhang H, Wang YH, Niu JM, Gan YJ, Zhong LR, Wang YF, Kapur A (2013). Evaluation of the value of fasting plasma glucose in the first prenatal visit to diagnose gestational diabetes mellitus in china. Diabetes Care.

[21] Werner EF, Pettker CM, Zuckerwise L, Reel M, Funai EF, Henderson J (2012). Screening for gestational diabetes mellitus: are the criteria proposed by the international association of the diabetes and pregnancy study groups cost-effective?. Diabetes Care.

[22] Meltzer SJ, Snyder J, Penrod JR, Nudi M, Morin L (2010). Gestational diabetes mellitus screening and diagnosis: a prospective randomised controlled trial comparing costs of one-step and two-step methods. BJOG.

[23] Cheung KW, Wong SF (2012). Gestational diabetes mellitus update and review of literature. Reproductive Sys Sex Disord.

[24] Cundy T, Ackermann E, Ryan EA (2014). Gestational diabetes: new criteria may triple the prevalence but effect on outcomes is unclear. BMJ.

[25] McIntyre HD, Sacks DA, Barbour LA, Feig DS, Catalano PM, Damm P, Mcelduff A (2016). Issues with the diagnosis and classification of hyperglycemia in early pregnancy. Diabetes Care.

[26] American Diabetes Association (2011). Standards of medical care in diabetes–2011. Diabetes Care.

[27] Duran A, Sáenz S, Torrejón MJ, Bordiú E, Del Valle L, Galindo M (2014). Introduction of IADPSG criteria for the screening and diagnosis of gestational diabetes mellitus results in improved pregnancy outcomes at a lower cost in a large cohort of pregnant women: the St.Carlos Gestational Diabetes Study. Diabetes Care.

[28] Wu ET, Nien FJ, Kuo CH, Chen SC, Chen KY, Chuang LM, Li HY, Lee CN (2016). Diagnosis of more gestational diabetes lead to better pregnancy outcomes: comparing the international association of the diabetes and pregnancy study group criteria, and the carpenter and coustan criteria. J Diabetes Investig.

[29] Diagnostic criteria and classification of hyperglycemia first detected in pregnancy. World Health Organization 2013, p.63; WHO/ NMH/ MND/ 13.2 http://apps.who.int/iris/bitstream/10665/85975/1/WHO_NMH_MND_13.2_eng.pdf .Accessed on 25 Dec 2015 24199271

[30] Australasian Diabetes In Pregnancy Society (ADIPS) Consensus Guidelines for the Testing and Diagnosis of Gestational Diabetes Mellitus in Australia. Available from: http://adips.org/downloads/ADIPSConsensusGuidelinesGDM-03.05.13VersionACCEPTEDFINAL.pdf. Accessed 25 Dec 2015.

[31] Hod M, Kapur A, Sacks DA, Hadar E, Agarwal M, Di Renzo GC, Cabero Roura L, McIntyre HD, Morris JL, Divakar H (2015). The International Federation of Gynecology and Obstetrics (FIGO) initiative on gestational diabetes mellitus: a pragmatic guide for diagnosis, management, and care. Int J Gynaecol Obstet.

[32] Criteria for the diagnosis of gestational diabetes in selected countries of the Americas. Final report of the Pan American Conference on Diabetes and Pregnancy. Available from: http://iris.paho.org/xmlui/bitstream/handle/123456789/28208/9789275118832_eng.pdf?sequence=1&isAllowed=y Last accessed 1^st^ May 2016

[33] Benhalima K, Mathieu C, Damm P, Van Assche A, Devlieger R, Desoye G, Corcoy R, Mahmood T, Nizard J, Savona-Ventura C, Dunne F (2015). A proposal for the use of uniform diagnostic criteria for gestational diabetes in Europe: an opinion paper by the European Board & College of Obstetrics and Gynaecology (EBCOG). Diabetologia.

[34] National Institute for Health and Care Excellence (NICE). Diabetes in pregnancy: management of diabetes and its complications from preconception to the postnatal period. NICE guidelines [NG3]. http://www.nice.org.uk/guidance/ng3/evidence. Published February 2015. Last accessed 1^st^ May 2016 25950069

[35] Screening for gestational diabetes. NICE guideline 3 Methods, evidence and recommendations. National Collaborating Centre for Women’s and Children’s Health. 2015. Available from: https://www.nice.org.uk/guidance/ng3/evidence/full-guideline-3784285 Last accessed 1^st^ May 2016

[36] Meek CL, Lewis HB, Patient C, Murphy HR, Simmons D (2015). Diagnosis of gestational diabetes mellitus: falling through the net. Diabetologia.

[37] Anjalakshi C, Balaji V, Balaji MS, Ashalata S, Suganthi S, Arthi T (2009). A single test procedure to diagnose gestational diabetes mellitus. Acta Diabetol.

[38] Chandramouli C. Rural urban distribution of population. Census of India. 2011. Available from: http://censusindia.gov.in/2011-prov-results/paper2/data_files/india/Rural_Urban_2011.pdf Last accessed 1^st^ May 2016

[39] Goonewardene M, Dias T (2013). Antenatal care: paradigm changes over the years. Ceylon Med J.

[40] Mohan V, Mahalakshmi MM, Bhavadharini B, Maheswari K, Kalaiyarasi G, Anjana RM (2014). Comparison of screening for gestational diabetes mellitus by oral glucose tolerance tests done in the non-fasting (random) and fasting states. Acta Diabetol.

[41] Pulkit Vij, Sujeet Jha SK, Gupta, Aneja Anjila, Mathur Rajani, Waghdhare Swati, et al. Comparison of DIPSI and IADPSG criteria for diagnosis of GDM: A study in a North Indian tertiary care center. Int J Diabetes Dev Ctries. 2015:1–4.

[42] Herath M, Priyantha T, Weerarathna UD (2015). Is non-fasting glucose challenge test sensitive enough to diagnose gestational diabetes mellitus?. Int Arch Med.

[43] Coustan DR, Widness JA, Carpenter MW (1986). Should the fifty-gram, one-hour plasma glucose screening test for gestational diabetes be administered in the fasting or fed state?. Am J Obstet Gynecol.

[44] Weiss PAM, Haeusler MCH, Kainer F, Purstner P, Haas J (1998). Towards universal criteria for gestational diabetes: relationships between 75 and 100 g glucose loads and between capillary and venous glucose concentrations. Am J Obstet Gynecol.

[45] Bhavadharini B, Mahalakshmi MM, Maheswari K, Kalaiyarasi G, Anjana RM, Deepa M (2016). Use of capillary blood glucose for screening for gestational diabetes mellitus in resource-constrained settings. Acta Diabetol.

